# I BRAZILIAN CONSENSUS ON MULTIMODAL TREATMENT OF COLORECTAL LIVER METASTASES. MODULE 2: APPROACH TO RESECTABLE METASTASES

**DOI:** 10.1590/0102-6720201600010003

**Published:** 2016

**Authors:** Héber Salvador de Castro RIBEIRO, Orlando Jorge Martins TORRES, Márcio Carmona MARQUES, Paulo HERMAN, Antonio Nocchi KALIL, Eduardo de Souza Martins FERNANDES, Fábio Ferreira de OLIVEIRA, Leonaldson dos Santos CASTRO, Rodrigo HANRIOT, Suilane Coelho Ribeiro OLIVEIRA, Marcio Fernando BOFF, Wilson Luiz da COSTA, Roberto de Almeida GIL, Tulio Eduardo Flesch PFIFFER, Fabio Ferrari MAKDISSI, Manoel de Souza ROCHA, Paulo Cezar Galvão do AMARAL, Leonardo Atem Gonçalves de Araújo COSTA, Tomas A. ALOIA, Luiz Augusto Carneiro D'ALBUQUERQUE, Felipe José Fernandez COIMBRA

**Affiliations:** 1 Brazilian Chapter of the International Hepato-Pancreato-Biliary Association - CB-IHPBA; 2 Brazilian Society of Surgical Oncology - SBCO; 3 Brazilian Society of Clinical Oncology - SBOC; 4 Brazilian College of Digestive Surgery - CBCD; 5 Brazilian College of Surgeons - CBC; 6 Americas Hepato-Pancreato-Biliary Association - AHPBA, Brazil; * Sociedade Brasileira de Radioterapia; ** Colégio Brasileiro de Cirurgiões

**Keywords:** Colorectal cancer, Liver metastases, Brazilian consensus, Resectable metastases, Metachronous metastases, Synchronous metastases, Missing metastases

## Abstract

**Background:**

Liver metastases of colorectal cancer are frequent and potentially fatal event in the evolution of patients.

**Aim:**

In the second module of this consensus, management of resectable liver metastases was discussed.

**Method:**

Concept of synchronous and metachronous metastases was determined, and both scenarius were discussed separately according its prognostic and therapeutic peculiarities.

**Results:**

Special attention was given to the missing metastases due to systemic preoperative treatment response, with emphasis in strategies to avoid its reccurrence and how to manage disappeared lesions.

**Conclusion:**

Were presented validated ressectional strategies, to be taken into account in clinical practice.

## Abstract (pt)

**Racional:**

As metástases hepáticas de câncer colorretal são evento frequente e potencialmente fatal na evolução dos pacientes.

**Objetivo:**

No segundo módulo desse consenso, foi discutido o manejo de metástases hepáticas ressecáveis.

**Método:**

Foi definido o conceito de metástases síncrônicas e metacrônicas, e ambos os cenários foram discutidos separadamente de acordo com as suas peculiaridades prognósticas e terapêuticas.

**Resultados:**

Foi dada especial atenção às missing metástases em resposta ao tratamento pré-operatório sistêmico, com ênfase em estratégias para evitar sua recorrência e como gerenciar as lesões desaparecidas.

**Conclusão:**

Foram apresentadas e validadas estratégias de ressecção em várias circunstâncias, para serem aplicadas na prática clínica.

## INTRODUCTION

Liver metastases of colorectal cancer (CRC) are common and life-threatening events in the evolution of patients with these malignancies. In this module is contextualized the possibilities of resection and results of various treatment modalities.

## METHOD

It was held discussion on the strategy on how and when to resect liver metastatic colorectal cancer, as well as the results of its application.

## RESULTS

## TOPIC 5 - Management of synchronous resectable disease

Synchronous colorectal liver metastases (CLM) are those diagnosed before, at the same time^1^ or up to six months after the detection of the primary tumor. This definition is heterogeneous in the literature since different publications adopt different times of disease progression of the colorectal tumor to characterize the synchronicity of hepatic lesions, which include intervals of four months^2^, six months^3^^,^ 4 and up to 12 months^5^^,^ 6. In this consensus, it was decided to adopt the interval of six months because this was the only that demonstrated impact on survival in prospective analysis^7^. 

This temporal definition led to two clinical scenarios in synchronous resectable disease, namely patients with primary in loco and those who received surgical treatment of the primary tumor but had metastatic disease detected within six months, likely the result of sub-optimal initial staging or rapid progression after the colorectal tumor treatment. The latter clinical scenario is particularly worrying when real disease progression is detected, especially during adjuvant treatment of the primary tumor, as it constitutes a poor prognostic factor with recommendation of upfront systemic therapy (or a change of regimen in adjuvant treatment) before liver resection^8^.

For patients with the primary tumor in loco, two distinctions are fundamental: if there are symptoms/risk of complications related to it during systemic treatment and whether the primary is a locally advanced mid/low rectal tumor and therefore demands neoadjuvant treatment.

The definition of symptomatic primary varies in the literature. The consensus adopted is the presence of obstructive syndrome (pain and abdominal distention with changed bowel habits caused by mass effect or luminal obstruction of the tumor) and active bleeding (enterorrhagia requiring blood transfusion) as parameters indicating surgical treatment of the primary tumor as the initial treatment^9^. The use of endoluminal prostheses appears as a feasible alternative to palliation of obstructive symptoms mainly as surgery with the aim of alleviating symptoms throughout systemic treatment is programmed^10^. For mid to low obstructive rectal tumors, the surgical option resides in temporary ostomies.

Another critical point in the initial assessment is the identification of patients who have higher risks of progressing with symptoms of the primary tumor during systemic treatment. For this reason, the incidence of emergency surgery seems low: between 3-15% in different series. The consensus adopted is that if an adult colonoscope device does not advance through the lesion, there would be a higher risk of obstructive symptons during systemic treatment. This does not necessarily mean that these patients, even if they do not exhibit clinical manifestations that fall within the definition of symptomatic primary mentioned above, must obligatorily receive surgical treatment of the primary before the start of systemic treatment, but it serves as a warning to maintain rigorous clinical follow-up in these cases^11^.

As for the initial approach of asymptomatic cases, in spite of the absence of prospective randomized data in the literature, the consensus points out that initial systemic treatment prioritizes occult micrometastatic disease, chemosensitivity testing of the disease to the proposed regimen, an increased rate of R0 resections and appears to improve recurrence-free survival (data extrapolated from a metachronous scenario), thus recommending this as standard conduct^12^. Chemotherapy regimens may include all agents for metastatic CRC (FOLFOX, XELOX, FOLFIRI and FOLFOXIRI), but the routine use of targeted therapy is not indicated in cases of clearly resectable liver metastases. Literature data suggest a deleterious effect of anti-EGFR therapy in this scenario and marginal benefit at the expense of increased toxicity, but also an increase in pathological response rates associated with anti-VEGF antibody to chemotherapy regimens^13^. In cases of patients with extensive liver disease (>4 nodes) or borderline resectability (where higher response rates may lead to a greater possibility of R0 resections), the consensus recommends individualized discussion in a multidisciplinary environment with the use of antiangiogenic therapy or anti-EGFR in RAS wild-type cases^14^^,^ 15. Preoperative treatment time should not exceed two to three months in order to reduce the risk of disappearance of liver lesions and prevent the occurrence of postoperative complications. The total recommended systemic treatment time is six months including the preoperative period and, if used, there is no indication for the maintenance of biological agents after complete resection of the primary and metastatic disease. The postoperative active regimens are based on fluoropyrimidines with or without oxaliplatin, since irinotecan has shown no benefit after hepatectomy for CRC liver metastases^16^.

Conversely, in patients with low-volume disease, complete preoperative staging and a simultaneous resection of the primary tumor and metastases with little risk of complication, the consensus is performing upfront surgery with postoperative chemotherapy for six months^11^.

In relation to the sequence of surgical treatment of primary tumors and metastases, there is great heterogeneity in the terms used in the literature and the consensus adopted uses the following definitions: classical or staged approach, where the resection of colorectal tumor and metastases is done in separate procedures and in the sequence: primary first and then metastases; Simultaneous approach in cases where the removal of the primary tumor and metastases is made ​​in a single surgical procedure; and the reverse approach, when liver metastases are resected first and in an isolated surgical procedure^17^. For asymptomatic primary colonic and high rectal tumors, the consensus is that following systemic treatment, classical and simultaneous approaches may be used depending on the extent of liver and colonic resection, while at the same time trying to avoid complex procedures. There is abundant literature attesting to the safety of simultaneous resection, including reducing morbidity related to cardiorespiratory complications^18^^,^ 19 ^,^ 20 ^,^ 21 ^,^ 22 ^,^ 23, but studies with the analysis of patients who exclusively underwent major hepatectomy showed significantly higher complication rates, thus showing this combination should be avoided^24^^,^ 25.

A distinction was made ​​for patients with primary tumors of the mid and low rectum because in cases of T3/T4 and/or N(+) lesions, neoadjuvant treatment must have radiation and chemotherapy included at some point of the its plan^26^. It is considered in the worse prognosis group, with greater intra- and mainly extrahepatic disease recurrence rate after an apparently curative treatment of the primary tumor and synchronous liver metastases^27^. The consensus recommendation is that these patients should receive as much treatment required for the primary and metastatic components of their disease, including effective upfront chemotherapy followed by neoadjuvant radio/chemotherapy of the rectal tumor with surgical resection, which can be made ​​in a simultaneous approach (straight+liver) or reverse (liver then straight - preferable to the classical approach as it does not expose the patient to an interval without systemic treatment with liver metastases in loco) depending on the complexity of each procedure.

## RECOMMENDATIONS

• Synchronous liver metastases are those detected before, concurrently or within six months of primary tumor diagnosis.

Agreement: 92%

Symptomatic primary tumors or at high risk of complications during systemic treatment should be resected and/or palliated prior.

Agreement: 94%

• For synchronous tumors with asymptomatic primary, consensus recommendation is to prioritize systemic treatment. Simultaneous upfront surgery and adjuvant chemotherapy is a valid option in cases of low risk of postoperative complications.

Agreement: 92%

• Primary rectal tumors with resectable liver metastases should receive as much treatment of both clinical conditions, including effective chemotherapy and neoadjuvant radio/chemotherapy of the primary when indicated. Case-by-case multidisciplinary discussion is strongly recommended for the definition of the treatment sequence.

Agreement: 87%

• The simultaneous approach is safe in capable patients and when at least one of resections (primary or metastases) is not complex. In all other cases the classical or reverse approaches should be preferred.

Agreement: 90%

## TOPIC 6 - Management of metachronous resectable disease

### Metachronicity

Treatment of CRC liver metastases can vary according to the time of diagnosis in relation to the primary tumor. In relation to this, the lesions can be described as synchronous or metachronous. Since the aim of this discussion is the treatment of metachronous lesions, the definition of metachronicity must first be established. 

Although some studies propose different time intervals, most of the series define metachronous liver metastases as those characterized in an adequate imaging test more than six months after diagnosis of the primary tumor. The main justification for the adoption of this interval is the rationale that lesions that recur until six months have similar biology to those diagnosed simultaneously. Some authors have questioned whether within the current scenario where the systemic treatment modalities are increasingly effective, the time interval should not be counted from the end of adjuvant treatment for the primary tumor^7^.

It should be mentioned that the presence of a prior imaging test is crucial to properly determine the time of metastasis diagnosis^4^.

### Initial resection

Metachronous lesions can be treated with upfront surgery followed by chemotherapy or in a multimodal treatment regimen with perioperative chemotherapy and surgery described in the EORTC 40983 study, in which 364 patients were randomized to receive six cycles of preoperative FOLFOX followed by surgery and another six adjuvant cycles, versus a control group treated with surgery alone. In this study there was a 7.3% gain of progression-free survival (35.4% vs 28.1%), but no significant gain in overall survival^12^. 

Some patients are better candidates for initial surgery, but there is no conclusive evidence to determine who these individuals are. The consensus from the European Registration of Cancer Care (EURECCA) recommends initial surgery for patients who have only metachronous lesions up to 2.0 cm because of the risk of complete radiological response if treatment starts with chemotherapy^28^. The purpose of this consensus is to encourage surgery at first for patients with favorable prognostic factors, but it was considered that there is no conclusive evidence to establish single factors that would select a specific group of individuals. In such cases, the recommendation is chemotherapy after resection for six months. 

### Surgery alone

In selected cases, individuals treated with upfront surgery may not be candidates to receive chemotherapy, thus being treated with surgery alone. These patients are those whose systemic treatment was associated with a higher risk of morbidity, such as the elderly or others who have compromised performance status at that time. Another scenario in which this treatment may be considered is when an excellent regimen of chemotherapy has recently been adopted and there is the onset of metachronous disease. This is known to be a worse prognosis scenario^29^.

### Choice of systemic treatment

The best evidence of multimodal treatment for CRC liver metastases is found in the previously mentioned EORTC 40983 study. From this data, both in patients undergoing perioperative chemotherapy and those who first receive surgery and subsequent chemotherapy, the chemotherapy of initial choice is oxaliplatin-based. Only in patients with metachronous lesions up to one year after adjuvant treatment with oxaliplatin is it considered not to repeat the drug.

 The use of biological agents associated with perioperative chemotherapy was investigated in the "New EPOC" study, in which the addition of cetuximab to chemotherapy was shown to lead to a reduction of overall and progression-free survival^13^. These findings contraindicate the association of this drug in this setting. 

 The use of other systemic treatment regimens associated with resection of liver metastases has also been investigated. Similar to anti-EGFR antibodies (cetuximab), the use of irinotecan did not improve survival compared to standard treatment^30^. The extrapolation of data to treat adjuvant micrometastatic disease of the primary tumor shows similar results for irinotecan^31^ and also bevacizumab^32^.

## RECOMMENDATIONS

• Definition of metachronicity: hepatic recurrence six months after onset of the primary tumo.

Agreement: 93%

• Initial surgery indication: Patients with favorable prognostic factors; there is no evidence that allows the individualization of this group of patients. These individuals should receive chemotherapy for six months after resection.

Agreement: 63%

• Consider surgery alone in individual candidates to initial surgery with compromised performance status or recurrence up to one year after adjuvant treatment with oxaliplatin.

Agreement: 71%

• When the decision is for perioperative treatment, the preferred chemotherapy regimen to be adopted is based on oxaliplatin and fluorouracil. After resection, the best evidence in the literature is to use isolated fluorouracil, although the consensus recommends adding oxaliplatin depending on the adjuvant treatment of the primary.

Agreement: 76%

• In the scenario of resectable disease, there is no evidence in the literature for the use of targeted therapy as well as irinotecan-based chemotherapy after R0 resection.

Agreement: 82%

## TOPIC 7 - Metastases with complete radiological response - what to do?

In recent years, with more effective chemotherapy regimens and increased use of preoperative chemotherapy in patients with liver metastases from colorectal cancer, came the concept of "missing metastases." This term defines CRC liver metastases that are not identified in the imaging methods after preoperative chemotherapy, not detected intraoperatively and that end up not being resected^33^.

 Obviously, the identification of metastases depends on the quality and extent of preoperative radiologic assessment. In general, "missing metastasis" occur is 5% of patients, but it may reach 36% in some series depending on the chemotherapy regimen used^34^. In up to 45% of patients, lesions are detected intraoperatively in sites that had disappeared on imaging tests^33^.

 True response is defined as the absence of viable tumor cells in the surgical specimen (pathologic complete response - pCR) or in the absence of local recurrence in a non-resected lesion after follow-up for at least 1 year^35^.

 Importantly, complete radiological response has limited predictive value for complete pathologic response. Likewise, the permanence of nodes in imaging tests does not necessarily mean that there are viable tumor cells in that lesion. Often fibrotic lesions with no viable cells appear in preoperative imaging tests as residual lesions after chemotherapy^34^.

 Literature data are very heterogeneous regarding correlation between radiological response and pCR. Imaging response is associated with pCR in 15-70% of cases. The recurrence rate for lesions that disappeared and were not resected can reach 74%^36^.

 There are no criteria to predict pCR. Some factors may help, such as normalization of CEA levels, absence of lesions on MRI and the use of intrahepatic arterial chemotherapy^37^. Factors that may help identify lesions with an increased risk of "disappearing" are: 1) use of intra-arterial chemotherapy; 2) prolonged duration of chemotherapy (>6 months); 3) lesions smaller than 2 cm^38^.

 The main imaging tests used to assess liver metastases after preoperative chemotherapy are CT, MRI, PET-CT and intraoperative ultrasound (IOUS). 

 CT is the most widely available test but has difficulty detecting nodules smaller than 1 cm and suffers interference from changes in the hepatic parenchyma after chemotherapy. MRI is the method that has better sensitivity to small nodules (<1 cm) and suffers less interference with post-chemotherapy changes, but it can be associated with higher costs and less availability at some centers. PET-CT is a costly method, has limited access and low sensitivity in this scenario due to decreased tumor metabolic activity after chemotherapy^39^.

 IOUS is the best test to assess hepatic lesions after preoperative chemotherapy. It provides extensive mobilization of the liver associated with visual inspection and palpation, aiding in locating deep and non-palpable lesions. Thus, it allows locating on average 30% (up to 67%) of the lesions that were not identified preoperatively^40^.

 In summary, it is recommended that preoperative chemotherapy for CRC liver metastases be used cautiously for lesions <2 cm and in cases of unresectable disease, its use be continued until the disease becomes resectable and not until maximum response. MRI is the modality of choice for the identification of small lesions after chemotherapy. IOUS, when available, is strongly recommended for "missing" lesions, as they are often found during surgical exploration with its use.

 Liver resection should be based on imaging tests immediately preceding chemotherapy treatment (neoadjuvant/conversion) and include the resection of segments containing the "missing" lesions when possible.

 When the "missing" lesion is present in a hepatic segment that cannot be resected (risk of impaired liver function), surgical treatment continues to be a good option, provided that all other identified lesions are resected or ablated.

### Recommendations

Preoperative chemotherapy for CRC liver metastases should be indicated with caution in the presence of lesions <2 cm due to the risk of disappearance and early re-assessment with image is recommended.

Agreement: 91%

In cases of unresectable disease, chemotherapy should be continued until the disease becomes resectable and not until maximal response.

Agreement: 91%

MRI - test of choice to identify small lesions after chemotherapy;

Agreement: 84%

Intraoperative ultrasound (IOUS) - strongly recommended ("missing" lesions are often found during surgical exploration/IOUS).

Agreement: 85%

Liver resection should be based on imaging tests immediately preceding chemotherapy treatment (neoadjuvant/conversion) and include the resection of the segments containing the "missing" lesions when possible.

Agreement: 89%

When the missing lesion is present in a hepatic segment that can not be resected (risk of impaired liver function), surgical treatment continues to be a good option, provided that all other identified lesions are resected or ablated. When not resected, missing metastases deserve close follow-up (CT/MRI every four months).

Agreement: 84%

## CONCLUSION

Resection strategies were presented and validated in several circumstances to be applied in clinical practice.
